# A Pro-Inflammatory Role of C5L2 in C5a-Primed Neutrophils for ANCA-Induced Activation

**DOI:** 10.1371/journal.pone.0066305

**Published:** 2013-06-13

**Authors:** Jian Hao, Chen Wang, Jun Yuan, Min Chen, Ming-Hui Zhao

**Affiliations:** 1 Renal Division, Department of Medicine, Peking University, First Hospital, Peking University Institute of Nephrology, Key Laboratory of Renal Disease, Ministry of Health of China, Key Laboratory of Chronic Kidney Disease Prevention and Treatment (Peking University), Ministry of Education, Peking-Tsinghua Center for Life Sciences, Beijing, China; 2 Renal Division, Department of Medicine, The Affiliated Hospital of Inner Mongolia Medical College Huhehot, Inner Mongolia, China; University of Leicester, United Kingdom

## Abstract

**Background:**

The complement system is crucial for the development of antineutrophil cytoplasmic antibody (ANCA)-associated vasculitis (AAV). In particular, C5a and its receptor on neutrophils, CD88, play a central role. The functional role of the second receptor of C5a, C5L2, remains unclear. In the current study, we investigated the role of C5L2 in C5a-primed neutrophils for ANCA-induced activation.

**Methods:**

The effect of blocking C5L2 by anti-human C5L2 blocking antibody were tested on respiratory burst and degranulation of C5a-primed neutrophils activated with ANCA, as well as on membrane-bound proteinase 3 (mPR3) and concentration of myeloperoxidase (MPO) in supernatant of C5a-primed neutrophils. An antagonist for CD88 was also employed.

**Results:**

Blocking C5L2 resulted in a significantly decreased MPO concentration in the supernatant of C5a-primed neutrophils. mPR3 expression increased from 209.0±43.0 in untreated cells to 444.3±60.8 after C5a treatment (*P*<0.001), and decreased to 375.8±65.44, 342.2±54.3 and 313.7±43.6 by pre-incubating blocking C5L2 antibody at 2.5 µg/ml, 5 µg/ml or 10 µg/ml (compared with C5a-priming group, *P*<0.001, *P*<0.001, and *P*<0.001), respectively. In C5a-primed neutrophils, subsequently activating with MPO-ANCA-positive IgG, the MFI value was 425.8±160.6, which decreased to 292.8±141.2, 289.7±130.0 and 280.3±136.4 upon pre-incubation with mouse anti-human C5L2 blocking antibody at 2.5 µg/ml, 5 µg/ml or 10 µg/ml (compared with C5a-primed neutrophils, for MPO-ANCA-positive IgG-induced activation, *P*<0.05, *P*<0.05, and *P*<0.05), respectively. Blocking C5L2 also resulted in significantly decreased C5a-primed neutrophils for PR3-ANCA-positive IgG-induced activation. Moreover, the lactoferrin concentration in the supernant significantly decreased in pre-incubation with anti-human C5L2 blocking antibody, compared with C5a-primed neutrophils induced by PR3- or MPO-ANCA-positive IgG.

**Conclusions:**

C5L2 may be implicated in the pro-inflammatory role in C5a-primed neutrophils for ANCA-induced activation.

## Introduction

Antineutrophil cytoplasmic antibody (ANCA)-associated vasculitis (AAV) includes granulomatosis with polyangiitis (GPA), microscopic polyangiitis (MPA) and eosinophilic granulomatosis with polyangiitis (EGPA) [1]. The two major antigens of ANCA are myeloperoxidase (MPO) and proteinase 3 (PR3) [1]. The pathogenesis of AAV has not been fully elucidated. *In vitro,* ANCAs activate primed neutrophils to undergo a respiratory burst and degranulation of granular constituents, which plays a direct pathogenic role in the development of vasculitic lesions [2]–[6].

The complement system is an important arm of innate immunity. In AAV, recent studies suggested that activation of the complement system was crucial for the disease development [7]–[13]. In particular, Schreiber et al. further found that recombinant C5a could dose-dependently prime neutrophils for ANCA-induced respiratory burst. The interaction between C5a and its receptor (C5aR, CD88) may compose an amplification loop and thus, plays a central role in ANCA-mediated neutrophil recruitment and activation [14].

C5a exerts its effects through two different receptors, i.e. CD88 and C5a receptor-like 2 (C5L2) [15], [16]. Most of the functional effects of C5a occur through CD88, which contributes to the initiation of acute inflammatory responses, such as chemotaxis, enzyme release and the respiratory burst [17], [18]. C5L2 is co-expressed with the CD88 on many kinds of cells including neutrophils. The function of C5L2 remains much more controversial, and thus is described as an “enigmatic” receptor by some authors [19], [20]. C5L2 might function as a default or modulating receptor for C5a, competing with CD88 for binding C5a [19], [21]. On the contrary, some other data suggested a functional role for C5L2 in certain diseases [22], [23]. The biological role of C5L2 appeared to be anti- or pro-inflammatory response to the anaphylatoxin in different disease settings [19]–[21]. However, the functional role of C5L2 in the pathogenesis of AAV is still unclear, and, to the best of our knowledge, has not been investigated yet. The current study investigated the role of C5L2 in C5a-primed neutrophils for ANCA-induced activation.

## Materials and Methods

### Preparation of IgG

ANCA-positive-IgG were prepared from plasma of patients with active MPO-ANCA- or PR3-ANCA-positive primary small vessel vasculitis. Plasma was filtered through a 0.22 μm syringe filter (Gelman Sciences, Ann Arbor, MI) and applied to a High-Trap-protein G column on an AKTA-FPLC system (GE Biosciences, South San Francisco, USA). Preparation of IgG was performed according to the methods described previously [24], [25]. We obtained written informed consent from all participants involved in our study. The research was in compliance of the Declaration of Helsinki and approved by the clinical research ethics committee of the Peking University First Hospital.

### Neutrophil Isolation

Neutrophils were isolated from heparinized venous blood of healthy donors by density gradient centrifugation on Lymphoprep (Nycomed, Oslo, Norway). Erythrocytes were lysed with ice-cold ammonium chloride buffer, and neutrophils were washed in Hanks balanced salt solution without Ca^2+^/Mg^ 2+^ (HBSS−/−; Chemical reagents, Beijing, China). Neutrophils were then suspended in HBSS with Ca^2+^/Mg^2+^ (HBSS+/+; Chemical reagents, Beijing, China) to a concentration of 2.5×10^6^ cells/ml and used for ANCA antigen translocation analysis, respiratory burst measurements and neutrophils degranulation.

### Membrane Expression of CD88 on Neutrophils after Pre-incubating Anti-human C5L2 Blocking Antibody

Flow cytometry was used to evaluate CD88 expression on neutrophils. In order to investigate the role of C5L2 in C5a-primed neutrophils activation, neutrophils were first incubated with mouse anti-human C5L2 blocking antibody (1D9-M12, Biolegend, San Diego, USA) [26]. Clone 1D9-M12 is well-known to block C5a by specifically binding to C5L2, but it does not react with CD88 [27]. However, in order to verify the anti-human C5L2 blocking antibody does not react with CD88 on neutrophils, cells were incubated with anti-human C5L2 blocking antibody at 2.5 μg/ml, 5 μg/ml or 10 μg/ml or buffer control for 30 min on ice. Next, cells were stained with a saturating dose of phycoerythrin (PE)-conjugated goat anti-human CD88 antibody (BD Biosciences, California, USA) for 30 min on ice. Fluorescence intensity of PE was analyzed using flow cytometry assessment of CD88 expression.

### Membrane Expression of PR3 on Neutrophils after Priming

Flow cytometry was used to evaluate PR3 expression on neutrophils. According the result of our previous study [11], the level of mPR3 expression was significantly higher on neutrophils primed with C5a at concentrations of 100 ng/ml than that before priming, and therefore, such concentration of C5a was employed in the following test unless indicated. In order to verify the pro-inflammatory role of CD88 in membrane expression of PR3 in C5a-primed neutrophils, neutrophils were first incubated with CD88 antagonist (NDT9513727, Tocris, Bristol, UK) (20 nM) for 30 min on ice. Nevertheless, in order to investigate the role of C5L2 in membrane expression of PR3 in C5a-primed neutrophils, neutrophils were first incubated with anti-human C5L2 blocking antibody for 30 min on ice. Then, cells were incubated with C5a at 100 ng/ml (Biovision, San Francisco, USA) or buffer control for 15 min at 37°C. This concentration of C5a was comparable to the circulating level of C5a in AAV patients in active stage, as described in our previous studies [12], [13]. All further steps were performed on ice and washing steps were carried out using HBSS +/+ containing 1% bovine serum albumin (BSA). Neutrophils were incubated with 0.5 mg/ml heat-aggregated goat IgG for 15 min to saturate Fcγ receptors. Next, cells were stained with a saturating dose of rabbit polyclonal antibody against human PR3 (Novus, Littleton, USA) or with an irrelevant IgG control antibody (Abcam, Cambridge, UK) for 30 min on ice. Neutrophils were then incubated with fluorescein isothiocyanate (FITC)-conjugated goat anti-rabbit antibody (BD Biosciences, California, USA) in the presence of 0.5 mg/ml heat-aggregated goat IgG. Fluorescence intensity of FITC was analyzed using flow cytometry assessment of PR3 expression. Samples were analyzed using a FACScan (Becton Dickinson, Germany). Neutrophils were identified in the scatter diagram, and data were collected from 10,000 cells per sample. The level of PR3 expression was calculated as MFI of specific binding of the isotype control antibody [25].

### Detection of MPO in the Supernatant of C5a-primed Neutrophils by ELISA

MPO in the supernatant of C5a-primed neutrophils was tested by ELISA using a commercial kit (USCNK, China). Cells were incubated with C5a (10, 100, 500 and 1000 ng/ml) (Biovision, San Francisco, USA) or buffer control for 15 min at 37°C. Neutrophils were pre-incubated with CD88 antagonist or mouse anti-human C5L2 blocking antibody (1D9-M12) for 30 min on ice. Then, cells were incubated with C5a (100 ng/ml) for 15 min at 37°C. Supernatant fluids were collected and used for ELISA analysis. The ELISA procedure of measuring MPO was as described previously [28]. In brief, the microtiter plate provided in this kit has been pre-coated with an antibody specific to MPO. Supernatant of neutrophils at dilutions of 1∶200 and standards were then added to the appropriate microtiter plate wells with a biotin-conjugated antibody preparation specific for MPO. Next, Avidin conjugated to horseradish peroxidase (HRP) was added to each microplate well and incubated. After TMB substrate solution was added, only those wells that contain MPO, biotin-conjugated antibody and enzyme-conjugated Avidin would exhibit a change in color. The enzyme-substrate reaction was terminated by the addition of sulphuric acid solution and the color change is measured spectrophotometrically at a wavelength of 450 nm. The concentration of MPO in the samples was then determined by comparing the O.D. of the samples to the standard curve.

### Measurement of Respiratory Burst by Oxidation of Dihydrorhodamine (DHR) to Rhodamine

We assessed the generation of reactive oxygen radicals using DHR as described previously [35]. The generation of reactive oxygen radicals was assessed using DHR. This method was based on the fact that reactive oxygen radicals cause an oxidation of the nonfluorescence DHR to the green fluorescence rhodamine. In brief, isolated neutrophils were gradually warmed to 37°C and incubated with 0.05 mM DHR123 (Sigma-Aldrich, Louis, USA) for 10 min at 37°C. Sodium azide (NaN_3_) (2 mM) was added in order to prevent intracellular breakdown of H_2_O_2_ by catalase. When indicated, cells were pre-incubated with CD88 antagonist or mouse anti-human C5L2 blocking antibody (1D9-M12) for 30 min on ice before the priming. Then, neutrophils were primed with 100 ng/ml C5a for 15 min at 37°C and incubated with patient-derived ANCA-positive-IgG (200 μg/ml) for 1 h at 37°C. The reaction was stopped by addition of 1 ml of ice-cold HBSS/1% BSA. Samples were kept on ice and analyzed using a FACScan. Neutrophils were identified in the scatter diagram, and data were collected from 10,000 cells per sample. The shift of green fluorescence in the FL-1 mode was determined. For each condition, the MFI (representing the amount of generated reactive oxygen radicals) was reported [24], [25].

### ANCAs Activated C5a-primed Neutrophils Degranulation

Lactoferrin, an iron binding multifunctional glycoprotein that was an abundant component of the specific granules of neutrophils [29], was considered as a biomarker of neutrophil degranulation [30]. Neutrophils were stimulated with C5a 100 ng/ml or buffer for 15 min followed by stimulation with MPO-ANCA-positive IgG or PR3-ANCA-positive IgG, or buffer control for 1 h, respectively. Neutrophils were pre-incubated with CD88 antagonist or mouse anti-human C5L2 blocking antibody (1D9-M12) for 30 min on ice before the priming. Supernatant fluids were collected and used for ELISA analysis. Lactoferrin in the neutrophils supernatant, as a measure of neutrophil degranulation, were tested by ELISA using a commercial kit (USCNK, China). In brief, the microtiter plate was pre-coated with a monoclonal antibody specific to lactoferrin. Cell supernatant at dilutions of 1∶500 and standards were then added to the appropriate microtiter plate wells with a biotin-conjugated polyclonal antibody preparation specific for lactoferrin. Next, avidin conjugated horseradish peroxidase (HRP) was added to each microplate well and incubated. Then a tetramethylbenzidine (TMB) substrate solution was added to each well. Only those wells that contained lactoferrin, the biotin-conjugated antibodies and enzyme-conjugated avidin would exhibit a change in color. The enzyme-substrate reaction was terminated by the addition of a sulphuric acid solution and the color change was measured spectrophotometrically at a wave-length of 450 nm±10 nm. The concentrations of lactoferrin in the samples were then determined by comparing the OD value of the samples to the standard curve.

### Statistical Analysis

Shapiro-Wilk test was used to examine whether the data was normally distributed. Quantitative data were expressed as means±SD (for data that were normally distributed) or median and range (for data that were not normally distributed) as appropriated. Differences of quantitative parameters between groups were assessed using the t test (for data that were normally distributed) or Mann-Whitney U test (for data that were not normally distributed) as appropriate. Differences were considered significant when *P*<0.05. Analysis was performed with SPSS statistical software package (version 16.0, Chicago, IL, USA).

## Results

### CD88 Expression on Neutrophils was not Significantly Influenced by Pre-incubation with Blocking C5L2 Antibody

**Figure 1:**
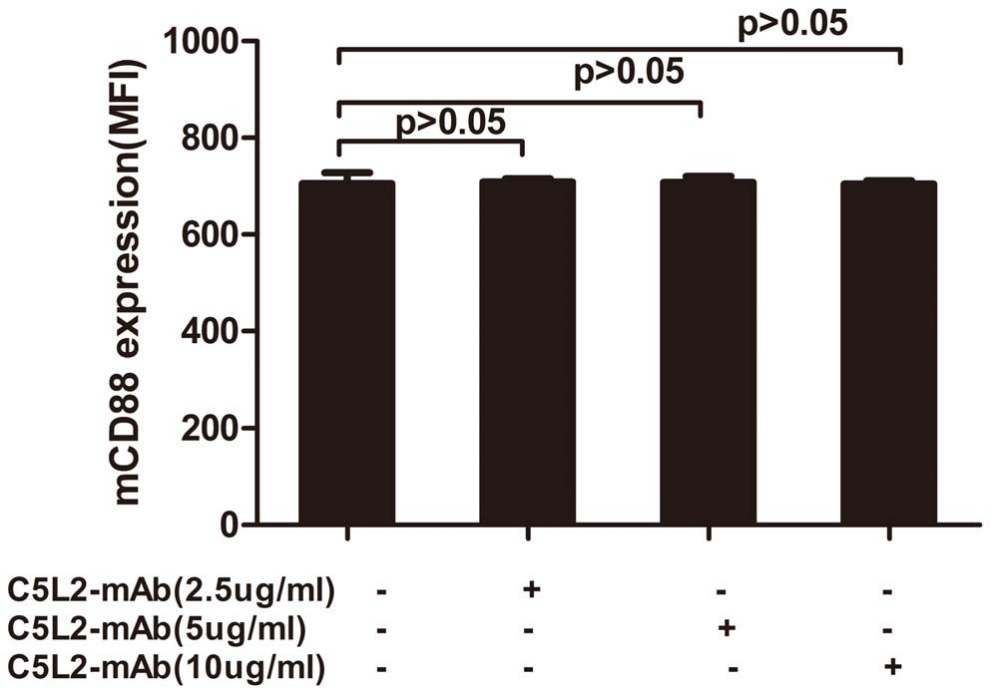
No effect of CD88 expression by pre-incubation with blocking C5L2 antibody.

Using flow cytometry, we showed parallel experiments that blocking C5L2 had no effect on membrane expression of CD88. No significant change of the expression of membrane-bound CD88 on neutrophils, expressed as the mean fluorescence intensity (MFI), after incubating with blocking C5L2 antibody at 2.5 µg/ml, 5 µg/ml or 10 µg/ml was observed (compared with groups without blocking C5L2 antibody, 708.7±7.1 vs. 705.7±22.1, 708.3±11.7 vs. 705.7±22.1, 704.3±6.8 vs. 705.7±22.1, *P*>0.05, *P*>0.05, and *P*>0.05, respectively) (Figure 1).

### C5a Increased MPO Concentration in the Supernatant of C5a-primed Neutrophils

**Figure 2:**
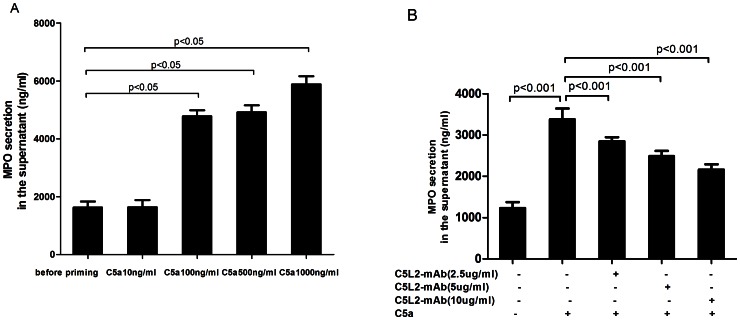
Blocking C5L2 decreased MPO concentration in the supernatant of C5a-primed neutrophils. MPO concentration was measured in the supernatant of C5a-primed neutrophils by ELISA. A: Human neutrophils were isolated and incubated with different concentrations of C5a for 15 min (n = 3). MPO concentration was measured in the supernatant of C5a-primed neutrophils and compared with non-stimulated cells. Bars denote means±SD of MPO concentration. B: Blocking C5L2 reduced MPO concentration in the supernatant of C5a-primed neutrophils. Bars represent mean±SD of MPO concentration, measured in the neutrophils supernatant of 6 independent experiments.

Neutrophils were incubated with different concentrations of C5a (10, 100, 500 and 1000 ng/ml), and MPO concentration in the supernatant of C5a-primed neutrophils was detected by ELISA. The concentration of MPO in the supernatant of C5a-primed neutrophils increased dose-dependently (1627.1±354.8ng/ml, 1629.6±433.5ng/ml, 4780.5±360.5ng/ml, 4916.8±419.5ng/ml, 5881.2±482.5ng/ml, for 0, 10, 100, 500 and 1000 ng/ml C5a, respectively). Compared with non-primed neutrophils, the concentration of MPO was significantly higher in the supernatant of neutrophils primed with C5a at concentrations of 100, 500 and 1000 ng/ml (*P*<0.05; *P*<0.05; *P*<0.05), respectively (Figure 2A).

### Blocking C5L2 Decreased MPO Concentration in the Supernatant of C5a-primed Neutrophils

MPO could be detected on the membrane of C5a-primed neutrophils but was mainly released to the extracellular medium. We showed parallel experiments that blocking C5L2 resulted in a decreased MPO concentration in the supernatant of C5a-primed neutrophils by ELISA. Since the MPO concentration in the supernatant of C5a-primed neutrophils was significantly higher on neutrophils primed with C5a at concentrations of 100 ng/ml than that before priming, such concentration of C5a was employed for the following test. MPO concentration increased from 1231.7±142.5 ng/ml in untreated cells to 3384.5±260.2 ng/ml in the supernatant of C5a-primed neutrophils (*P*<0.001) and decreased to 2848.9±100.0ng/ml, 2490.2±125.2 ng/ml and 2163.1±130.0 ng/ml by pre-incubating blocking C5L2 antibody at 2.5 µg/ml, 5 µg/ml or 10 µg/ml (compared with C5a-induced group, *P*<0.001, *P*<0.001, and *P*<0.001), respectively (Figure 2B).

### Blocking C5L2 Decreased the Expression of Membrane-bound PR3 (mPR3) on C5a-primed Neutrophils

**Figure 3:**
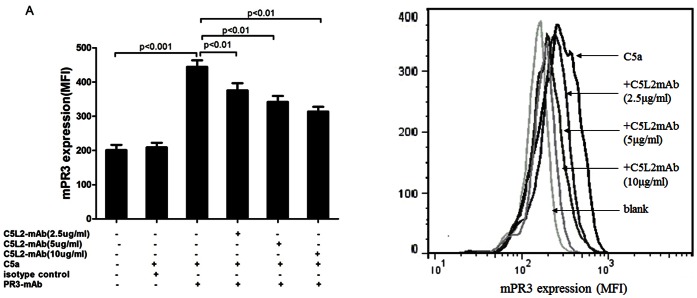
Blocking C5L2 decreased C5a-primed neutrophils expression of membrane-bound PR3 (mPR3). A: Human neutrophils were isolated and incubated with C5a (100 ng/ml) for 15 min (n = 11). Neutrophils were pre-incubated with the blocking C5L2 mAb for 30 min. Bars represent mean±SD of repeated measurements on neutrophils of 10 independent experiments and donors. B: a representative histogram of effects of the blocking C5L2 mAb at 2.5 µg/ml, 5 µg/ml or 10 µg/ml on translocation of PR3 upon C5a priming.

Expression of mPR3 on neutrophils of 10 healthy donors was analyzed. Neutrophils were incubated with 100 ng/ml of C5a, and mPR3 expression was determined by flow cytometry. Since we previously found increases in mPR3 expression were much stronger during neutrophils priming compared with MPO [11], we only explored the variation of C5a-mediated translocation of PR3 to the cell surface when blocking C5L2. Using flow cytometry, we showed parallel experiments that blocking C5L2 resulted in a decreased C5a-induced translocation of PR3. mPR3 expression increased from 209.0±43.0 in untreated cells to 444.3±60.8 after C5a treatment (p<0.001), and decreased to 375.8±65.44, 342.2±54.3 and 313.7±43.6 by pre-incubating blocking C5L2 antibody at 2.5 µg/ml, 5 µg/ml or 10 µg/ml (compared with C5a-priming group, *P*<0.01, *P*<0.01, and *P*<0.01), respectively (Figure 3). Together, these experiments indicated that C5L2 involved in C5a-mediated translocation of PR3 from the intracellular granules to the cell surface.

### Blocking C5L2 Decreased C5a-primed Neutrophils for ANCA-induced Respiratory Burst

**Figure 4:**
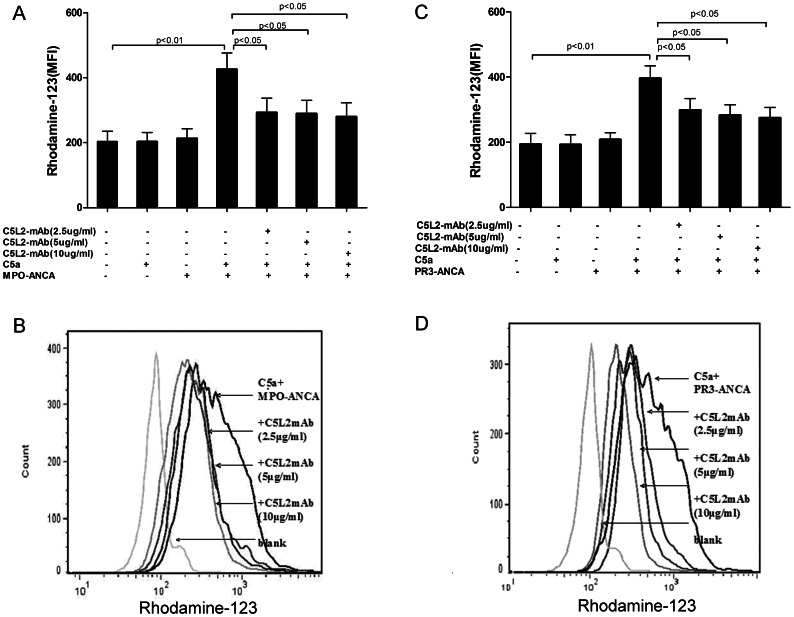
Blocking C5L2 decreased C5a-primed neutrophils for ANCA-induced respiratory burst. Neutrophil respiratory burst induced by patient-derived MPO-ANCA (A) or PR3-ANCA (C) was measured by conversion of dihydrorhodamine-123 (DHR-123) to rhodamine-123 in C5a-primed cells in the presence and absence of blocking C5L2 mAb. Bars represent mean±SD of 4 MPO-ANCA and 2 PR3-ANCA IgG preparations, each measured on neutrophils of 10 independent experiments and donors. B and D: representative histograms showing effects of the blocking C5L2 mAb at 2.5 µg/ml, 5 µg/ml or 10 µg/ml on MPO-ANCA positive IgG or PR3-ANCA positive IgG-induced respiratory brust in C5a-primed neutrophils.

We studied the variation of C5a-primed neutrophils for ANCA-induced respiratory burst when blocking C5L2. ANCAs-IgG were prepared from 4 patients with active MPO-ANCA-positive vasculitis and 2 patients with active PR3-ANCA-positive vasculitis, respectively. Compared with non-primed neutrophils, the MFI value increased significantly in C5a-primed neutrophils activated with MPO-ANCA-positive IgG and PR3-ANCA-positive IgG (425.8±160.6 vs. 213.5±95.2, *P*<0.01; 415.9±102.2 vs. 207.8±68.4, *P*<0.01, respectively) (Figure 4A and C). No obvious respiratory burst activity was observed with C5a or ANCA-IgG alone. We next investigated the variation of C5a-primed neutrophils for ANCA-induced respiratory burst when blocking C5L2. Neutrophils were pre-incubated with mouse anti-human C5L2 blocking antibody before primed with C5a and the subsequent stimulated with ANCA. Pre-incubation of neutrophils with mouse anti-human C5L2 blocking antibody decreased oxygen radical production in C5a-primed neutrophils induced by ANCA-positive IgG from patients. In C5a-primed neutrophils, subsequently activating with MPO-ANCA-positive IgG, the MFI value was 425.8±160.6, which decreased to 292.8±141.2, 289.7±130.0 and 280.3±136.4 upon pre-incubated with mouse anti-human C5L2 blocking antibody at 2.5 µg/ml, 5 µg/ml or 10 µg/ml (compared with C5a-primed neutrophils for MPO-ANCA-positive IgG-induced activation, *P*<0.05, *P*<0.05, and *P*<0.05), respectively. For PR3-ANCA IgG, the MFI value was 415.9±102.2 in C5a-primed neutrophils, which decreased to 299.0±110.0, 282.9±102.3 and 274.8±101.0 upon pre-incubation with anti-human C5L2 blocking antibody (compared with C5a-primed neutrophils for PR3-ANCA-positive IgG-induced activation, *P*<0.05, *P*<0.05 and *P*<0.05), respectively (Figure 4).

### Blocking C5L2 Decreased C5a-primed Neutrophils for ANCA-induced Degranulation

**Figure 5:**
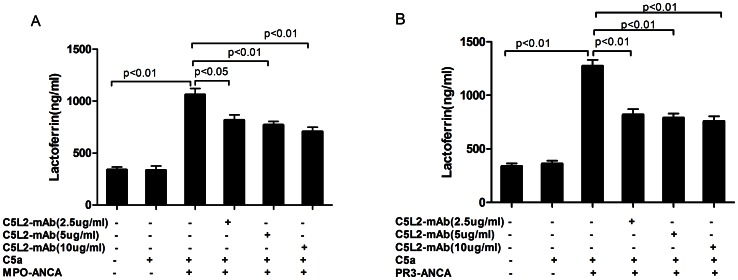
Blocking C5L2 decreased C5a-primed neutrophils for ANCA-induced degranulation. ANCA-induced neutrophil degranulation was determined by measuring the lactoferrin concentrations in the supernatant. Blocking C5L2 reduced ANCA-induced lactoferrin release. Bars represent mean±SD of 4 MPO-ANCA and 2 PR3-ANCA preparations, measured on neutrophils of 10 donors.

Degranulation was determined by measuring the lactoferrin concentration in the C5a-primed for ANCA-induced neutrophil supernatant. Pretreatment with anti-human C5L2 blocking antibody reduced MPO-ANCA-positive IgG-induced and PR3-ANCA-positive IgG-induced lactoferrin release. The lactoferrin concentration increased from 338.8±26.7 ng/ml in the non-primed neutrophils supernatant to 1261.7±60.0 ng/ml in C5a-primed neutrophils induced by MPO-ANCA-positive IgG (*P*<0.01), and decreased to 817.3±50.3 ng/ml, 771.5±33.4 ng/ml, and 706.3±43.1 ng/ml upon pre-incubation with anti-human C5L2 blocking antibody at 2.5 µg/ml, 5 µg/ml or 10 µg/ml (compared with C5a-primed neutrophils induced by MPO-ANCA-positive IgG, *P<*0.05, *P<*0.01, and *P<*0.01), respectively. In C5a-primed neutrophils induced by PR3-ANCA-positive IgG, the lactoferrin concentration in the supernatant increased from 174.2±57.5 ng/ml in untreated cells to 338.8±26.7 ng/ml (*P*<0.01), which decreased to 819.7±52.7 ng/ml, 789.1±41.1 ng/ml and 758.5±46.2 ng/ml upon pre-incubation with anti-human C5L2 blocking antibody at 2.5 µg/ml, 5 µg/ml or 10 µg/ml (compared with C5a-primed neutrophils induced by PR3-ANCA-positive IgG, *P*<0.01, *P*<0.01 and *P*<0.01), respectively (Figure 5).

### Blocking CD88 Decreased ANCA Antigen Translocation, ANCA-induced Respiratory Burst and Degranulation in C5a-primed Neutrophils

**Figure 6:**
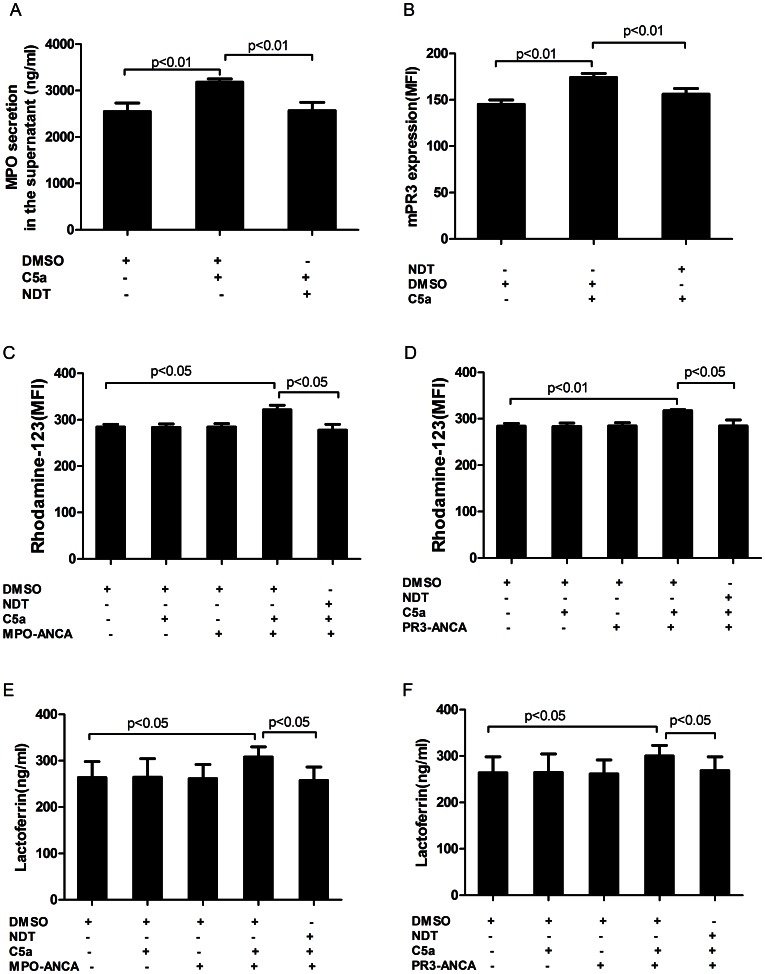
Blocking CD88 decreased ANCA antigen translocation, ANCA-induced respiratory burst and degranulation in C5a-primed neutrophils. A: blocking CD88 reduced MPO concentration in the supernatant of C5a-primed neutrophils. Bars represent mean±SD of MPO concentration, measured in the neutrophils supernatant of 6 independent experiments. B: blocking CD88 decreased C5a-primed neutrophils expression of membrane-bound PR3 (mPR3). C and D: blocking CD88 decreased C5a-primed neutrophils for ANCA-induced respiratory burst. E and F: blocking CD88 decreased C5a-primed neutrophils for ANCA-induced degranulation. Bars represent mean±SD of repeated measurements on neutrophils of 6 independent experiments and donors.

MPO concentration increased from 2486.4±310.8 ng/ml in untreated cells to 3225.0±218.3 ng/ml in the supernatant of C5a-primed neutrophils (*P*<0.01), and decreased to 2580.4±248.8 ng/ml by pre-incubating CD88 antagonist (NDT9513727) at 20 nM (compared with the C5a-primed group, *P*<0.01) (Figure 6A). Using flow cytometry, we showed parallel experiments that blocking CD88 resulted in a decreased C5a-induced translocation of PR3. mPR3 expression increased from 134.2±21.1 in untreated cells to 176.0±4.8 after C5a treatment (*P*<0.01), and decreased to 153.8±13.7 by pre-incubating CD88 antagonist at 20 nM (compared with the C5a-primed group, *P*<0.01) (Figure 6B). Pre-incubation of neutrophils with CD88 antagonist decreased oxygen radical production in C5a-primed neutrophils induced by ANCA-positive IgG from AAV patients. In C5a-primed neutrophils, subsequently activating with MPO-ANCA-positive IgG, the MFI value was 383.6±86.4, which decreased to 276.4±15.6 upon pre-incubation with CD88 antagonist at 20 nM (compared with C5a-primed neutrophils, for MPO-ANCA-positive IgG-induced activation, *P*<0.05). For PR3-ANCA-positive IgG, the MFI value was 370.2±79.0 in C5a-primed neutrophils, which decreased to 270.8±46.9, upon pre-incubation with a CD88 antagonist (compared with C5a-primed neutrophils for PR3-ANCA-positive IgG-induced activation, *P*<0.05) (Figure 6C and D). Pretreatment with CD88 antagonist reduced MPO-ANCA-positive IgG-induced and PR3-ANCA-positive IgG-induced lactoferrin release. The lactoferrin concentration in the supernatant increased from 255.1±23.9 ng/ml in the non-primed neutrophils supernatant to 312.4±43.0 ng/ml in C5a-primed neutrophils induced by MPO-ANCA-positive IgG (*P*<0.05), and decreased to 253.3±25.5 ng/ml upon pre-incubation with CD88 antagonist at 20 nM (compared with C5a-primed neutrophils induced by MPO-ANCA-positive IgG, *P<*0.05). In C5a-primed neutrophils induced by PR3-ANCA-positive IgG, the lactoferrin concentration in the supernatant increased from 263.8±30.4 ng/ml in untreated cells to 300.8±29.3 ng/ml (*P*<0.05), which decreased to 273.3±30.5 ng/ml upon pre-incubation with CD88 antagonist at 20 nM (compared with C5a-primed neutrophils induced by PR3-ANCA-positive IgG, *P*<0.05) (Figure 6E and F).

## Discussion

ANCA-induced neutrophils respiratory burst and degranulation are important contributors to the development of ANCA-associated vasculitis. Recent studies, both in the mouse model and in human, suggested that complement activation is involved in the pathogenesis of AAV [7]–[13]. Among the complement activation products, C5a is a strong chemoattractant for neutrophils (reviewed by Guo et al. [31]). C5a, together with its receptor on neutrophils, CD88, seems to play a vital role in the pathogenesis of AAV, demonstrated by both animal and in vitro studies [14]. In this study, we found that pretreatment with CD88 antagonist reduced C5a-primed neutrophils for ANCA-induced activation, which verified the pro-inflammatory role of CD88 in AAV. However, the pathophysiological role of C5L2, a putative “default” receptor for C5a, is still unclear in AAV.

In our previous study, we found that the increase of membrane-bound MPO expression was much lower than membrane-bound PR3 expression after incubation with C5a [11]. MPO was mainly released into the extracellular medium after C5a priming. This result was in line with some other studies [14], [28], [32]–[35].

In the current study, in order to investigate the pathophysiological role of C5L2 in AAV, the anti-human C5L2 blocking antibody, Clone 1D9-M12, was employed to block C5L2. C5L2 binds C5a with similarly high affinity as CD88 [19]; however, Clone 1D9-M12 is known to specifically blockade C5a binding to C5L2 [26]. Pre-incubated with the anti-human C5L2 blocking antibody (1D9-M12) in C5a-primed neutropuils, the effect of C5a binding to CD88 did not change [19], [26]. Similarly, we found that pre-incubated with the anti-human C5L2 blocking antibody (1D9-M12) had no effect on membrane expression of CD88 in our study.

The most important finding in the current study was that blocking C5L2 decreased C5a-primed neutrophils for ANCA-induced respiratory burst and degranulation, which relied on blocking the priming effect of C5a. It suggested that C5L2 may play a pro-inflammatory role in C5a-primed neutrophils for ANCA-induced activation. C5L2 binds C5a with similarly high affinity as CD88 [36], [37]. By binding to CD88, C5a activated neutrophils through the recruitment of G protein, which resulted in the increasing intracellular Ca^2+^ together with MAPK and Akt activation [38]. C5a or up-regulated CD88 expression has been implicated in the pathogenesis of many autoimmune diseases [20]. However, C5L2 is uncoupled from G proteins due to the lack of critical intracellular amino acid motifs [37]. This lack of signaling after binding with its ligands suggested that C5L2 may function as a decoy receptor regulating the bioavailability of C5a [37]. In contrast to many previous studies, our data suggested that C5L2 might be a functional receptor rather than a default receptor.

Mechanistically, the process of C5L2 in C5a-mediated signal transduction was less clear. It was found that C5L2 had a limited ability to transduce signals, which was likely to remove active complement fragments from the extracellular environment [38]. C5a treatment alone strongly induced ERK1/2 activation in wild-type neutrophils, whereas the induction of activated ERK1/2 in response to C5a was decreased in C5L2^−/−^ neutrophils. Moreover, C5L2 has been reported to mediate β-arrestin redistribution [19], [27].

Recently, several studies indicated that C5L2 has functional roles in regulating inflammation [39], such as in asthma [40] and sepsis [23]. Chen et al [22] found that C5L2 is involved in the pathogenesis of asthma-like airway hyper-responsiveness and inflammation. In addition, macrophages molecules 1 (Mac-1) induction [22] and high mobility group box 1 (HMGB1) release [23] was impaired in C5L2^−/−^ neutrophils in response to C5a alone compared with wild-type cells.

In conclusion, our studies with blocking monoclonal antibody to human C5L2 have demonstrated C5L2 play a pro-inflammatory role in C5a-primed neutrophils for ANCA-induced activation. Blockade C5L2 might limit inflammatory damage caused by ANCA-activated neutrophils.
